# Tuberculosis Case Finding Cascade and Treatment Outcomes among Male Inmates in Two Prisons in Zimbabwe

**DOI:** 10.1155/2020/5829471

**Published:** 2020-07-08

**Authors:** Alois Mandizvidza, Riitta A. Dlodlo, Palanivel Chinnakali, Hamufare Dumisani Mugauri, Freeman Dube, Evidence Gaka, Joseph Nembaware, Shepherd Nyambi, Blessing Masunungure, Davison Garauzive

**Affiliations:** ^1^Harare Central Prison, Zimbabwe Prison and Correctional Service, Harare, Zimbabwe; ^2^International Union against Tuberculosis and Lung Disease (The Union), Paris, France; ^3^Departments of Preventive and Social Medicine, Jawaharlal Institute of Postgraduate Medical Education & Research (JIPMER), Puducherry, India; ^4^AIDS and TB Unit, Ministry of Health and Child Care, Harare, Zimbabwe; ^5^National Aids Council (NAC), Harare, Zimbabwe

## Abstract

**Design:**

A cohort study using programmatic data was undertaken to assess TB diagnostic cascade in one of the study prisons for 2018. Treatment outcomes among male inmates with TB were assessed over a period of four years, in two study prisons.

**Results:**

A total of 405 (11%) inmates with presumptive TB were identified, and 370 (91%) of these were evaluated for TB, mostly using rapid molecular testing of sputum specimens. Twenty-five inmates were diagnosed with TB resulting in a prevalence of 649/100,000 population. Of these, 16 (64%) were started on treatment. Nine (36%) were lost to follow-up before treatment initiation. From 2015 to 2018, 280 adult male inmates with TB were started on treatment. Of these, 212 (76%) had pulmonary disease that was bacteriologically confirmed. Almost all (276/280, 99%) had known HIV status, 65% were HIV-infected, and 80% of these were on antiretroviral treatment. The TB treatment success rate (cured or treatment completed) was recorded for 209 (75%) inmates, whilst 14 (5%) died and 11 (4%) were lost to follow-up. The frequency of unfavourable treatment outcomes (death, lost to follow-up, and not evaluated) was higher (54%) among inmates ≥ 60 years than those in the age group of 45-59 years (17%).

**Conclusion:**

The findings revealed a threefold burden of TB in prisons, compared with what is reported by national survey. To decrease transmission of TB bacilli, it is essential to promote efforts that address missed opportunities in the TB diagnostic cascade, prompt treatment initiation, and ensure that tracking and documentation of treatment outcomes for all inmates are intensified.

## 1. Introduction

Worldwide prisons are recognised as major congregate institutions with an extraordinarily high burden of tuberculosis (TB), including multidrug-resistant TB, and inadequate resources for health and TB services [1]. It has been estimated that TB in prisons may reach levels that are up to 100 times higher than those among civilian populations. In selected countries, prison populations may account for 25% of national TB burden [2]. Late diagnosis, inadequate treatment, overcrowding, poor ventilation, and repeated prison transfers contribute to rampant transmission of TB bacilli. Also, HIV infection, malnutrition, alcohol and substance abuse, and other comorbidities are common among inmates and contribute to development of active TB disease [2].

Studies suggest that 50% of inmates with TB may remain undiagnosed and therefore untreated and not notified, creating a source for continuous transmission [3]. Modest TB treatment success of 66% and high proportions of inmates who are lost to follow-up have been reported [4]. Effective TB activities in prisons protect inmates, prison staff, visitors, and community at large [2]. End TB Strategy recognises the need for strengthening TB care and prevention in prisons to attain its ambitious targets [5].

Since the 1990s, Zimbabwe has been devastated by an HIV-associated TB epidemic. In 2017, the estimated TB prevalence was 344 per 100,000 population with an estimated HIV prevalence of 63% among TB patients [6]. A study carried out in 2015-2016 in 24 prisons in the country in a sample of 691 inmates found presumptive TB in 112 (16%) persons who were investigated, and four were found to have TB resulting in TB prevalence of 575/100,000 population [7]. To our knowledge, no studies of TB treatment outcomes among prison inmates in the country have been performed.

This study is aimed at assessing the TB case finding cascade in Harare Central Prison and the programmatic treatment outcomes among inmates in Harare Central Prison and Chikurubi Maximum Prison. The first objective included assessment, among male inmates at admission to Harare Central Prison, to determine the number and proportion of (i) presumptive TB, (ii) presumptive TB evaluated, (iii) bacteriologically confirmed and clinically diagnosed TB, and (iv) initiated on TB treatment. The second objective was to describe, among inmates with TB and initiated on treatment at Harare Central and Chikurubi Maximum Prisons, (i) treatment outcomes and (ii) sociodemographic and disease characteristics associated with unfavourable outcomes.

### 1.1. Setting

#### 1.1.1. General Setting

Zimbabwe is one of the southern African countries experiencing an HIV-associated TB epidemic with an estimated prevalence of 344/100,000 population [6].

### 1.2. Background

#### 1.2.1. Zimbabwe Prisons and Correctional Service

The Zimbabwe Prisons and Correctional Service (ZPCS) has a total of 46 established and 24 satellite prisons with an official holding capacity of approximately 17,000 inmates. Most prisons were built several decades ago and have not benefited from renovations resulting in dilapidated buildings with poor ventilation. The prisons are overcrowded, and some of them hold double the number of inmates compared to their recommended maximum holding capacity.

ZPCS has a health directorate responsible for health care services for inmates and staff. There are forty-six established clinics and four prison hospitals staffed by nurses, doctors, and other health professionals. TB diagnostic services are available at Chikurubi Maximum (Harare) and Khami Maximum (Bulawayo) Prison hospitals. One 4-modular XpertMTB/RIF® (Cepheid, Sunnyvale, CA, USA) platform is situated in the laboratory of Chikurubi Maximum Prison where specimens from prisons in Harare are processed. TB treatment is available in all prison clinics. All sputum smear microscopy examinations are done at the National Microbiology Reference Laboratory (NMRL).

#### 1.2.2. Study Sites

Chikurubi Maximum and Harare Central Prisons are among the largest prisons in the country with inmate capacities of 2500 and 2042, respectively. Their health facilities include 30- and 28-bedded hospitals in Chikurubi and Harare Central Prisons, respectively. In the former, there is a laboratory and a radiology facility. Both prison hospitals have an outpatient department attending to 20-40 people a day and a clinic for HIV care. The health staff consists of two medical officers, one clinical officer, 125 nurses, and support staff that include pharmacy, nutrition, dental, laboratory, and rehabilitation officers.

Upon admission to prison, convicted individuals undergo a general medical examination that includes a TB symptom screen that is also undertaken when inmates develop symptoms suggestive of TB during their stay. It includes cough of any duration, weight loss, fever, night sweats, and haemoptysis in the last 12 months and neck swellings. A person with any one of these symptoms is considered a person with presumptive TB and evaluated with the following: sputum examination using XpertMTB/RIF® test and/or chest radiography. All persons with presumptive TB are also offered HIV counselling and testing. They are registered in the presumptive TB register, and the investigation results are documented. Upon diagnosis of TB (bacteriologically confirmed or clinically diagnosed, including extrapulmonary disease), inmates are isolated and started on treatment consisting of six months of isoniazid and rifampicin and, during the initial 2-month intensive phase, of pyrazinamide and ethambutol. This treatment is given daily with support by clinic nurses who administer the fixed-dose combination medication.

The patients are notified to the local health authorities. Monitoring of TB treatment response and recording and reporting follow the national guidelines [8]. If the inmate with TB is released before completion of TB treatment, the relevant local authority is informed through a telephone call so that treatment can be continued at the health facility closest to the person's residence. The NTP transfer out form is also filled in [9]. TB focal nurses and environmental health technicians stationed at the prisons register inmates found to have TB in the TB treatment register and maintain the details of treatment, including the treatment outcomes, in this register following the standardised definitions as listed in the national TB manual [9].

## 2. Methods

### 2.1. Study Design

This was a retrospective cohort study using routinely available data on TB services provided by the prison health services.

### 2.2. Study Population

We included men aged 18 years and above admitted to Harare Central Prison from 1 January 2018 to 31 December 2018 for assessing the diagnostic cascade. For evaluating treatment outcomes, we included male inmates aged 18 years and above with drug-susceptible TB and started on TB treatment at Harare Central and Chikurubi Maximum Prisons from 1 January 2015 to 31 December 2018.

### 2.3. Data Variables, Sources of Data, and Data Extraction

For assessment of TB case finding cascade, information on people with presumptive TB, people with their sputum specimens collected, sent to the laboratory, and with results received, and HIV status were extracted from the presumptive TB register. Laboratory registers were used to record, register, and track specimens received as well tracking of sputum results. We obtained the total number of individuals admitted to the prison from the admission register. Data on the number of people with TB by year, their characteristics, type of TB, and TB treatment outcomes were extracted from TB treatment registers.

### 2.4. Analysis and Statistics

The data were entered using EpiInfo™ software (7.2.3.1, CDC, Atlanta, GA, USA), and analyses were performed in Stata software (version 22.0, Stata Corp LLC, College Station, TX, USA). A descriptive analysis was performed using frequencies and proportions. TB treatment outcomes were described as proportions with 95% CI and categorised into favourable outcomes (cured, completed treatment, and treatment success as combined) and unfavourable outcomes (died, lost to follow-up, failed treatment, and not evaluated). Association of age, residence, site (Harare/Chikurubi), and clinical characteristics (type of TB, site of TB, and HIV status) with unfavourable outcomes was assessed using the chi-squared test and unadjusted relative risk. Multivariable regression analysis (log-binomial model) was done to assess the independent effects of exposure variables on treatment outcomes. All the exposure variables were included in the model. Levels of significance were set at 5%.

### 2.5. Ethics Approval

The study protocol was reviewed and approved by the Medical Research Council of Zimbabwe (MRCZ/E/252) and The Union Ethics Advisory Group, Paris, France (42/19). The prisons' management gave permission for the access and review of the records of the inmates with presumptive and diagnosed TB.

## 3. Results

**Figure 1:**
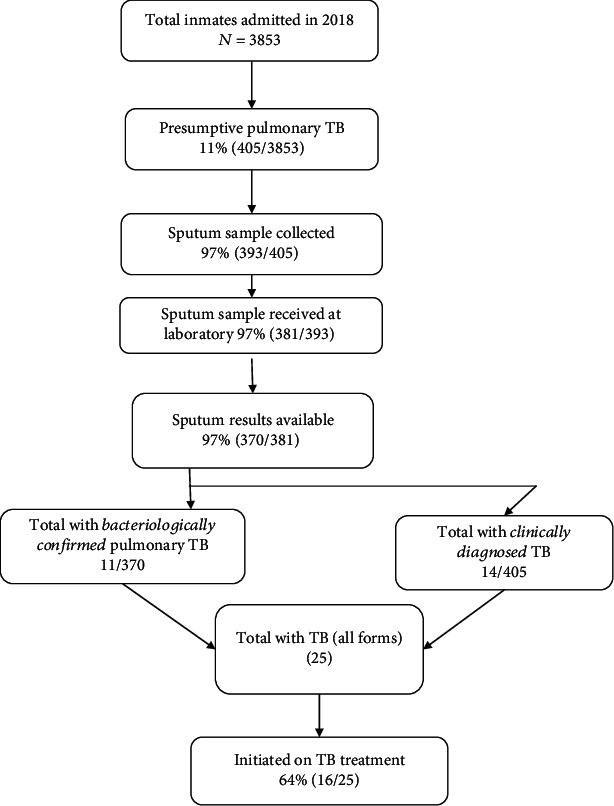
Flow chart describing the number of adult inmates admitted and evaluated for tuberculosis in Harare Central Prison, Zimbabwe, 2018.

In 2018, a total of 3853 inmates were admitted to Harare Central Prison. Of these, 405 (11%) screened positive for TB symptoms and were registered in the presumptive TB register (Figure 1). A total of 313 (77%) people with presumptive TB were aged 25-44 years and 154 (38%) were HIV-infected (not shown). A total of 370 (91%, 370/405) people with presumptive disease completed TB evaluation, and all but one had a sputum XpertMTB/RIF test. Ten people had *Mycobacterium tuberculosis* detected on XpertMTB/RIF (all rifampicin-susceptible), and one person was diagnosed through sputum microscopy resulting in a positivity rate of 3% (11/370) and a notification rate of 285 cases of bacteriologically confirmed pulmonary TB/100,000 population. In addition, there were 14 clinically diagnosed TB, including one person with extrapulmonary TB. It followed that the TB (all forms) notification rate was 649/100,000 population. Sixteen (64%, 16/25) people were started on TB treatment. The remaining nine (36%, 9/25) could have been transferred or released before commencement on treatment.

From 2015 to 2018, a total of 280 adult male inmates admitted to Harare Central Prison and Chikurubi Maximum Prison were initiated on TB treatment. The median (interquartile range) age was 35 (30-42) years. Their characteristics are presented in Table 1. Two hundred and twelve (75%) were reported to have pulmonary disease, and 121 (43%) had bacteriologically confirmed TB. HIV status was unknown for four (1.4%) of these individuals. Of those with known HIV status, 65% (180/276) were HIV-positive, and of these, 144 (80%, 144/180) were reported to be on antiretroviral treatment.

The TB treatment success rate (cured or treatment completed) was recorded for 209 (75%, 95% CI 69%-80%) individuals. The outcome “not evaluated” was the most frequently (46, 16%) reported unfavourable outcome followed by death (14, 5%) (Table 2).

The factors associated with TB treatment outcomes are described in Table 3. The occurrence of unfavourable outcomes (death, loss to follow-up, and “not evaluated”) was higher (54%) among inmates ≥ 60 years than those in the age group of 45-59 years (17%, *P* = 0.030).

## 4. Discussion

This is one of the first studies to report on the TB case finding cascade and treatment outcomes in correctional services in Zimbabwe.

Our study in one large prison in Harare showed that 405 (11%) of admissions had presumptive TB and that 12 (3%) people with presumptive TB were lost during TB evaluation. A high TB notification rate was revealed. Pretreatment loss to follow-up was 36%. Assessment of TB treatment outcomes in this and another large prison revealed a treatment success rate (75%) that was lower than the rate (83%, 2017) reported for the country and for male TB patients (90%, 2017) in Harare, and almost one out of four people with TB did not have a TB treatment outcome [9]. The treatment success rate could have been affected by frequent transfers.

The strengths of this study included the use of routinely available programmatic data that were likely to be representative of operational realities in this setting. In addition, the study report adhered to the Strengthening the Reporting of Observational Studies in Epidemiology (STROBE) guidelines [10]. Limitations included the use of the programmatic registers and the recorded data: frequently, data were missing and they could have been inaccurate. Due to unavailability of recorded dates in the registers, we were unable to assess the time taken to make a diagnosis and start TB treatment in those found to have TB that may play an important role in reducing transmission in congregate settings.

The losses (9%) in TB case finding cascade in Harare Central Prison compared favourably with the losses reported for the entire country (24%) in 2018 [9]. The sputum test positivity rate (3%) among inmates on admission and during the stay of inmates was found to be lower than the proportion (6%) among all people with presumptive TB in the country in the same year [9].

The TB prevalence found by our study was threefold compared with the national estimate, and it was in line with the prevalence reported by an earlier assessment [7]. However, it was considerably lower than the rate reported by a study in DRC and was at the lower end of the prevalence reported by a review of studies published from 24 sub-Saharan African countries [11]. This review reported an HIV prevalence of 2%-35% among inmates with TB, and the prevalence we found was considerably higher (64%).

High rates of prediagnosis and pretreatment loss to follow-up have been reported from both the urban and rural areas of Zimbabwe, and these were in line with the finding by our study [9].

Previous studies have reported TB treatment success rates among inmates that ranged from 46% in South Africa and 48% in Uganda to 80% in Ethiopia [12]. The treatment success (75%) in our study compared favourably but was lower than the national rate (83%) in Zimbabwe in 2017 [9]. The proportion of loss to follow-up and/or people with nonevaluated outcomes was reported to be high ranging from 15% in Ethiopia and 16% in our study to 43% in Uganda and 51% in South Africa [3].

This study has the following programmatic implications. First, whilst the rate of presumptive TB was high, it is critical that the missed opportunities in TB case finding cascade in this setting with congestion of inmates and old facilities with a shortage of isolation rooms and inadequate ventilation are reduced. This can be achieved through regular review of the presumptive TB register and ensuring regular specimen transportation to the laboratory and communication of sputum test results back to the clinical staff. It is also possible that some clinically diagnosed TB was missed due to weak access to radiology services.

Second, though the considerable pretreatment loss to follow-up could be partially explained by transfers of inmates to other prisons, the correctional services should set up a system that facilitates tracking of people diagnosed with TB, especially those with infectious forms of the disease. Refresher training for health professionals responsible should be arranged to stress the importance of close observation of sputum test and other investigation results and prompt initiation of appropriate treatment if TB is diagnosed. This is essential to decrease continued transmission of TB bacilli within the correctional services making them “safer”.

Third, complete and accurate documentation is critical for data-driven monitoring of TB care and prevention services. It can be enhanced through health staff training in active local use of routinely available TB data for decision-making and ensuring that support supervision is data-driven.

In conclusion, our study findings are suggestive of disproportionate TB burden in the two prisons in the country. The findings have also revealed programmatic gaps, such as losses in the TB case finding cascade and considerable pretreatment loss to follow-up that should be brought to zero. Concerted efforts to ensure that all inmates with TB have a treatment outcome that is correctly documented should also be ensured.

## Figures and Tables

**Table 1:** Sociodemographic and clinical characteristics of adult inmates with tuberculosis admitted at Harare Central Prison and Chikurubi Maximum Prison, Zimbabwe, 2015-2018.

Characteristics	Total	Percentage
Total	280	
Age groups in years		
18-29	63	22.5
30-44	168	60.0
45-59	36	12.9
≥60	13	4.6
Residence		
Urban	151	53.9
Rural	122	43.6
Not recorded	7	2.5
Site of TB		
Pulmonary	212	75.7
Extrapulmonary	15	5.4
Not recorded	53	18.9
Classification by sputum status		
Bacteriologically confirmed	121	43.2
Clinically diagnosed	135	48.2
Not recorded	24	8.6
Classification by history of TB treatment		
New	227	81.1
Previously treated	49	17.5
Not recorded	4	1.4
HIV and ART status		
HIV negative	96	34.3
HIV positive and on ART	144	51.4
HIV positive and not on ART	29	10.4
HIV positive and ART status unknown	7	2.5
HIV status unknown	4	1.4

TB: tuberculosis; ART: antiretroviral therapy.

**Table 2:** Tuberculosis treatment outcomes among adult inmates admitted at Harare Central Prison and Chikurubi Maximum Prison, Zimbabwe, 2015-2018.

Treatment outcomes^∗^	Number	Percentage with 95% CI
Cured	123	43.9 (38.0-50.0)
Treatment completed	86	30.7 (25.4-36.5)
Died	14	5.0 (2.8-8.2)
Lost to follow-up	11	4.0 (2.0-7.0)
Failure	0	—
Not evaluated	46	16.4 (12.3-21.3)
Total	280	100

^∗^As defined in the national TB manual, Zimbabwe, 2016. CI: confidence interval.

**Table 3:** Factors associated with tuberculosis treatment outcomes among adult inmates admitted at Harare Central Prison and Chikurubi Maximum Prison, Zimbabwe, 2015-2018.

Characteristics	Total	Unfavourable^∗^ outcome, *n* (%)	Unadjusted RR (95% CI)	Adjusted RR^†^ (95% CI)	*P* value^‡^
Total	280	71 (25.4)	—	—	
Age groups in years					
18-29	63	17 (27.0)	1.62 (0.70-3.73)	1.56 (0.67-3.62)	0.298
30-44	168	41 (24.4)	1.46 (0.67-3.18)	1.48 (0.68-3.22)	0.328
45-59	36	6 (16.7)	1	1	
≥60	13	7 (53.9)	3.23 (1.33-7.84)	2.80 (1.1-6.9)	**0**.**030**
Residence					
Urban	151	40 (26.5)	1.08 (0.72-1.62)	1.19 (0.78-1.81)	0.420
Rural	122	30 (24.6)	1	1	
Not recorded	7	1 (14.3)	0.58 (0.09-3.66)	0.75 (0.13-4.29)	0.748
Site of TB					
Pulmonary	212	49 (23.1)	1	1	
Extrapulmonary	15	3 (20.0)	0.87 (0.31-2.45)	0.77 (0.26-2.27)	0.637
Not recorded	53	19 (35.9)	1.55 (1.00-2.39)	1.41 (0.82-2.4)	0.210
Classification by sputum status					
Bacteriologically confirmed	121	22 (18.2)	1		
Clinically diagnosed	135	39 (28.9)	1.59 (1.00-2.52)	1.36 (0.79-2.32)	0.265
Not recorded	24	10 (41.7)	2.29 (1.25-4.19)	1.99 (1.04-3.81)	**0**.**038**
Classification by history of TB treatment					
New	227	56 (24.7)	1	1	
Previously treated	49	14 (28.6)	1.16 (0.70-1.90)	1.07 (0.66-1.72)	0.793
Not recorded	4	1 (25.0)	1.01 (0.18-5.61)	0.87 (0.10-7.8)	0.901
HIV and ART status					
HIV negative	96	25 (26.0)	1	1	
HIV positive and on ART	144	36 (25.0)	0.96 (0.62-1.49)	1.04 (0.66-1.64)	0.871
HIV positive and not on ART	29	7 (24.1)	0.93 (0.45-1.92)	0.88 (0.40-1.91)	0.739
HIV positive and ART status unknown	7	2 (28.6)	1.10 (0.32-3.71)	1.22 (0.39-3.83)	0.734
HIV status unknown	4	1 (25.0)	0.96 (0.17-5.42)	1.34 (0.15-12.2)	0.794

RR: relative risk; CI: confidence interval; TB: tuberculosis; ART: antiretroviral therapy. ^∗^Unfavourable outcomes include death, loss to follow-up, and “outcome not evaluated.” ^†^Adjusted RR is based on the multivariable model (log-binomial regression), and all exposure variables were included in the model. ^‡^*P* values are from multivariable analysis, and *P* values < 0.05 are highlighted in bold.

## Data Availability

The programmatic data used to support the findings of this study are available from the corresponding author upon request.

## Additional Points

*Open-Access Statement*. In accordance with WHO's open-access publication policy for all work funded by WHO or authored/coauthored by WHO staff members, the WHO retains the copyright of this publication through a Creative Commons Attribution IGO License (http://creativecommons.org/licenses/by/3.0/igo/legalcode) which permits unrestricted use, distribution, and reproduction in any medium provided the original work is properly cited.
